# Case Report: Amyloid-Producing Odontogenic Tumor With Pulmonary Metastasis in a Spinone Italiano—Proof of Malignant Potential

**DOI:** 10.3389/fvets.2020.576376

**Published:** 2020-09-29

**Authors:** Callie Blackford Winders, Cynthia M. Bell, Stephanie Goldschmidt

**Affiliations:** ^1^College of Veterinary Medicine, University of Minnesota, St. Paul, MN, United States; ^2^Specialty Oral Pathology for Animals, Geneseo, IL, United States

**Keywords:** amyloid producing odontogenic tumor, odontogenic tumor, neoplasia, dogs, pathology—head and neck neoplasms

## Abstract

A 1-year-old male Spinone Italiano dog was treated for an amyloid-producing odontogenic tumor on the right maxilla with a cytoreductive surgery followed by a definitive radiation protocol. Six years later, the dog presented for a new mass on the rostral mandible as well as a lung nodule without recurrence of the original maxillary tumor. Both the mandibular mass and the lung nodule were histologically confirmed to be amyloid-producing odontogenic tumor based on the appearance of sheets and cords of the odontogenic epithelium disrupted by amorphous extracellular amyloid. This case illustrates the metastatic potential for amyloid-producing odontogenic tumor in dogs and asynchronous occurrence of multiple APOTs in the oral cavity.

## Introduction

The amyloid-producing odontogenic tumor (APOT) is an uncommon odontogenic neoplasm in dogs and cats. Based on previous case reports, it occurs most commonly in older dogs with a predilection for small breeds, but large clinicopathologic studies are lacking (1–6). APOT has also been reported in a goat, prairie dog, and Bengal tiger (7–9). This tumor was previously compared to the calcifying epithelial odontogenic tumor (CEOT) in humans based on microscopic similarity, but current consensus is that APOT in animals has no true analog in humans.

Histologically, APOTs are characterized by the presence of the odontogenic epithelium with extracellular deposition of amyloid-like material (1–6). Previous investigators have shown that the material has positive immunolabeling targeting ameloblast proteins (amelogenin, sheathlin, and ameloblastin) and suggested that this tumor would be better referred to as amyloid-producing ameloblastoma (5).

Similar to ameloblastoma, this tumor is generally regarded as benign yet locally invasive into surrounding bone and teeth (1–6). It is widely believed that these tumors carry no metastatic potential, and there have been no reports of metastasis in any animal species. Recently, a case of ameloblastic carcinoma arising from an APOT in a miniature dachshund was reported (10). Here, we describe the first case of a dog with a confirmed metastatic APOT, which was discovered 6 years after excision of the original oral mass and also identified concurrently with a secondary APOT within the oral cavity.

## Case History

**Figure 1:**
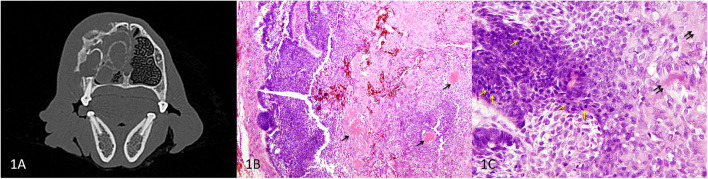
Primary APOT of the rostral maxilla, dog. **(A)** Axial CT image of the skull at the level of 106 showing the maxillary mass. **(B)** HE, 40×. In the peripheral region of the mass (left of image), odontogenic epithelial cells are organized in sheets and cords. Lower cellularity in the central portion of the mass is the result of extracellular deposits of amorphous eosinophilic material (presumed amyloid) that multifocally coalesce to resemble osteoid/dentinoid matrix (arrows). **(C)** HE, 400×. The neoplastic epithelial cells have basilar to acanthomatous morphology. Architecture is less distinct where cells have abundant cytoplasm and produce extracellular amyloid (double arrows). Mitotic figures (yellow arrows) are common among the basal cells although nuclear atypia was low to moderate.

Initial presentation of the APOT was in a 1-year-old castrated male Spinone Italiano dog. There was a firm, nonpainful mass over his right rostral maxilla measuring 7 cm craniocaudal × 4 cm mediolateral × 2.5 cm dorsoventral that had been noted for approximately 1 month prior to presentation. A CT scan of the skull was performed using a 64-slice helical CT scanner (Toshiba Aquilion 64, Canon Medical Systems, Tustin, CA). Pre- and post-contrast transverse images were acquired at 0.5-mm slice thickness and 0.3-mm slice interval and subsequently reconstructed into 2-mm slice thickness by 2-mm slice interval. Ioversol (Optiray 350, Guerbet LLC, Bloomington, IN) was administered intravenously at a 2-mL/kg body weight for contrast images. Imaging revealed an expansile, multiloculated lesion with cortical lysis as well as irregular periosteal reaction affecting the right maxilla from the canine tooth to the fourth premolar (104–108) (Figure 1A). The right mandibular and retropharyngeal lymph nodes were enlarged, of uniform attenuation, and of relatively uniform enhancement. The primary differentials for the right rostral maxillary lesion, based on the age of the patient, cystic appearance of the lesion, and close association with the teeth, included odontogenic cyst and cystic odontogenic tumor with non-odontogenic neoplasia being less likely. Thoracic radiographs showed no evidence of metastatic disease at this time.

A dorsal rhinotomy was performed for a wedge incisional surgical biopsy. Amyloid-producing odontogenic tumor was diagnosed, and a cytoreductive right segmental maxillectomy was performed from mesial 104 to distal 107. The surgical pathology confirmed the diagnosis of APOT based on proliferation of the odontogenic epithelium in association with extracellular eosinophilic material that was interpreted as amyloid (Figure 1B). Mitotic figures were present at a rate of 25 per 10 high power fields (2.37 mm^2^), although nuclear atypia was mild to moderate (Figure 1C). Most surgical margins were clean although the dorsal margin was considered to be equivocal in the microscopic sections. Therefore, the dog underwent postoperative definitive radiation therapy (22 fractions × 2.4 Gy).

**Figure 2:**
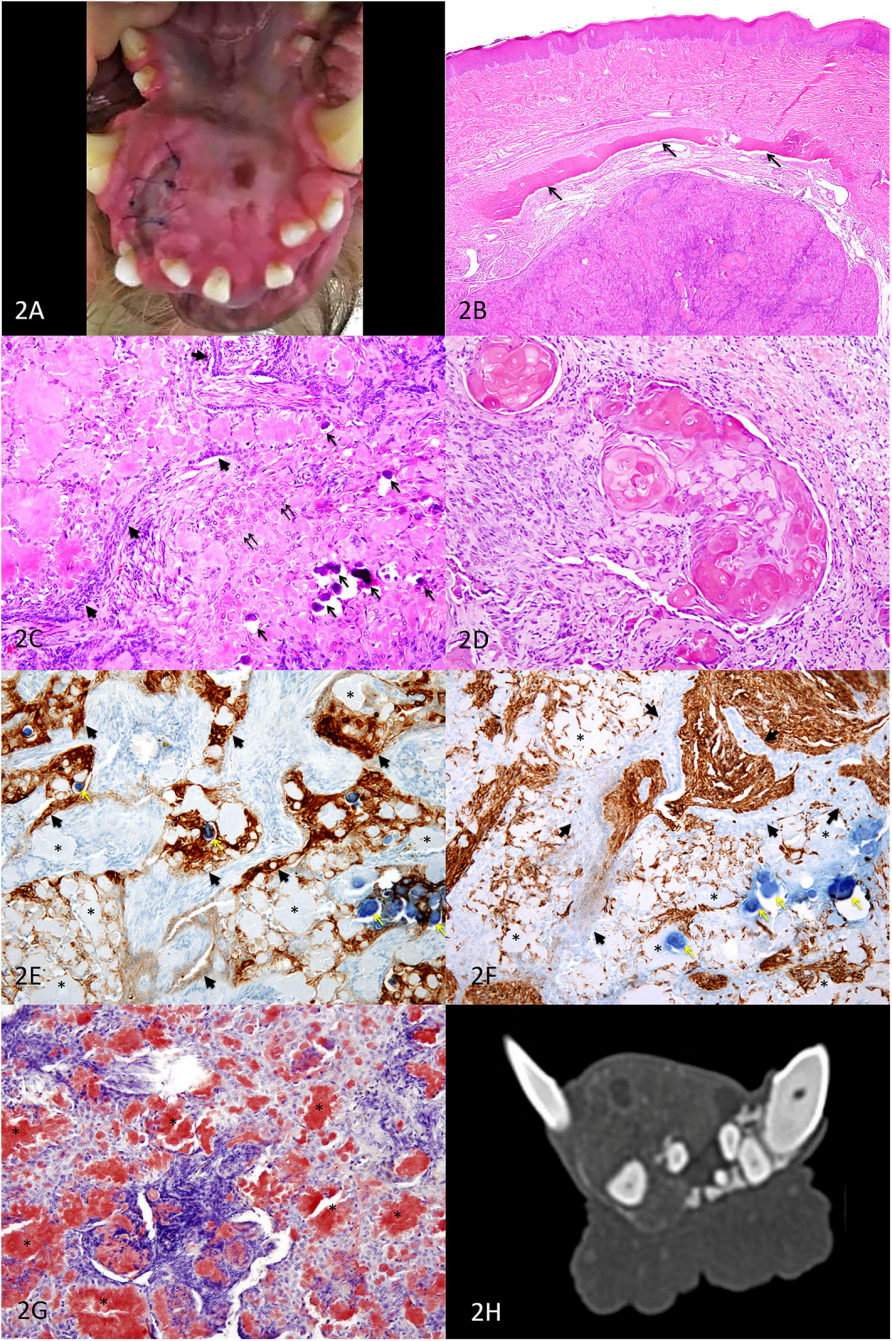
Second APOT on the rostral mandible, dog. **(A)** A mass lingual to the right mandibular incisors and canine expands the rostral mandible. **(B)** HE, 40×. An expansile intraosseous mass displaced a rim of atrophied mandibular bone (arrows) and the overlying gingiva is unremarkable in this section. **(C)** HE, 200×. Cords of small basilar epithelial cells (arrowheads) and sheets of larger polygonal cells (double arrows) are occasionally distinct although much of the tissue architecture is disrupted by abundant extracellular eosinophilic amyloid, which is multifocally mineralized (arrows). The clear space surrounding the mineralized foci is artifact of processing. **(D)** HE, 200×. In some areas of the tumor, neoplastic epithelial cells had ghost cell morphology. The abundant keratinized cytoplasm can be difficult to differentiate from lighter pink amyloid. **(E)** IHC for pancytokeratin AE1/AE3, DAB chromogen, 200×. Sheets and cords of epithelial cells (arrowheads) have moderate to strong positive labeling of the cytoplasm, including cells that are among the amyloid. The amyloid (*) is negative, and the mineralized foci are dark blue (yellow arrows). **(F)** IHC for vimentin, DAB chromogen, 200×. Sheets and cords of epithelial cells (arrowheads) have minimal or no positive labeling of the cytoplasm, in contract to the supporting connective tissues. The amyloid (*) is negative, although some cells surrounding the amyloid have positive labeling. The mineralized foci are dark blue (yellow arrows). **(G)** Congo red, 200×. Extracellular amyloid deposits are congophilic (*), having deep red positive staining. **(H)** Axial CT image of the mandibular mass.

Six years later, there was no recurrence of the right maxillary mass and the dog re-presented to the University of Minnesota for assessment of a new firm, round, expansile gingival mass causing displacement of adjacent teeth on the right rostral mandible (2 cm craniocaudal × 3 cm mediolateral; Figure 2A). On presentation, a mesenchymal tumor, possibly radiation induced such as fibrosarcoma or osteosarcoma (11), was considered most likely. However, histopathology from an incisional wedge biopsy revealed a second APOT.

Microscopically, an intraosseous epithelial neoplasm expanded within bone and was associated with abundant amorphous extracellular material (Figure 2B). Palisading cords of small polygonal cells resembled dental lamina, and diffuse sheets of epithelial cells were disrupted by the abundant amyloid, which was rarely calcified (Figure 2C). There were occasional nests of “ghost cells,” which are large anucleate squamous epithelial cells with keratinization (Figure 2D). No mitotic figures were observed in 10 high-power fields (2.37 mm^2^), and nuclear atypia was mild. Because the abundance of amyloid so disrupted architecture of the neoplasm, immunohistochemistry with pancytokeratin AE1/AE3 (Figure 2E) and vimentin (Figure 2F) was useful in distinguishing between the neoplastic epithelial population and the supporting stromal cells. The amyloid was strongly congophilic (Figure 2G).

For staging purposes, a CT scan of the head, neck, thorax, and abdomen was performed using a 64-slice helical CT scanner. Pre- and post-contrast transverse images of the skull, neck, thorax, and abdomen were obtained at 1.0-mm slice thickness and 0.8-mm slice interval and subsequently reconstructed into 2-mm slice thickness by 2-mm slice interval. Ioversol (Optiray 350, Guerbet LLC, Bloomington, IN) was administered intravenously at a 2-mL/kg body weight for contrast images. Post-contrast sequences were obtained in the arterial, venous, and delayed phase. CT lymphangiography was performed with 1 ml of ioversol combined with 1 ml of sterile saline for sentinel lymph-node mapping. It was elected to perform sentinel lymph-node mapping and include the thorax and abdomen in the preoperative imaging due to age of the dog and history of multiple previous therapies performed for treatment of the initial oral mass.

Images of the head and neck revealed a predominantly soft tissue-attenuating, heterogeneously contrast-enhancing mass containing amorphous mineralization and several internal non- to minimally enhancing cavitations at the most rostral aspect of the right mandible measuring approximately 19 mm (H) × 22.7 mm (W) × 25 mm (L) (Figure 2H). CT scan confirmed no local recurrence of the APOT previously excised on the right maxilla. The mandibular and medial retropharyngeal lymph nodes were normal in size and appearance, and the abdominal scan revealed no abnormalities.

**Figure 3:**
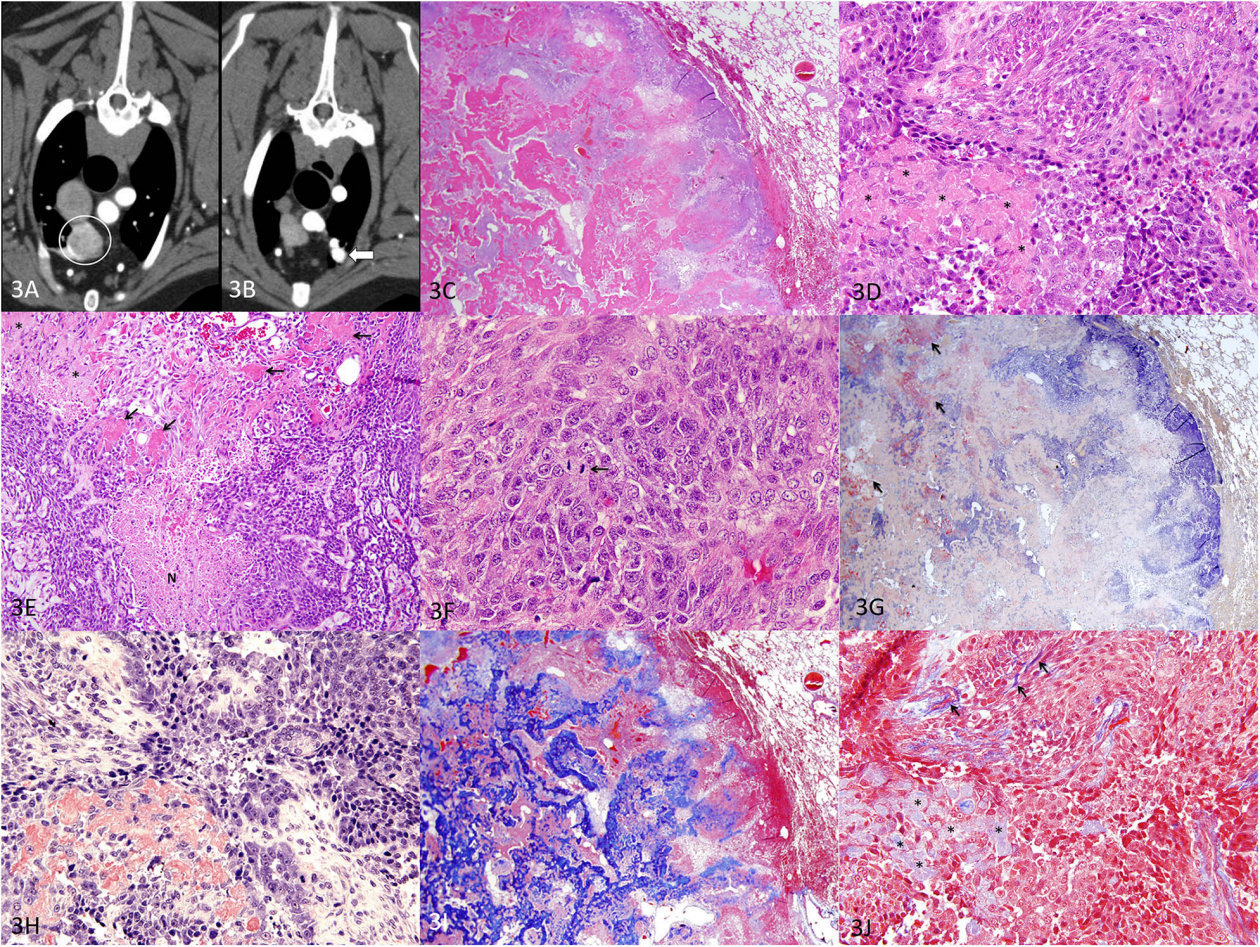
Metastatic APOT in the lung, dog. **(A)** Axial CT image of the right cranial lung lobe mass indicated by the circle. **(B)** Axial CT image of the nodule noted in the left cranial lung lobe indicated by the arrowhead. **(C)** HE, 20×. The mass (left of image) is well demarcated, and adjacent lung tissue (right of image) is minimally compressed with intact architecture. The lung tumor has areas of purple cellularity intermingled with abundant eosinophilic material that coalesces into trabeculae. **(D)** HE, 400×. As in the original oral APOT), sheets of cells are disrupted by extracellular amyloid (*). **(E)** HE, 200×. The lung tumor had plexiform branching of cords of small polygonal cells with areas of necrosis (N). The amyloid (*) was less intensely eosinophilic yet distributed in the same pattern as the smallest deposits of brightly eosinophilic, osteoid-like matrix (arrows). **(F)** HE, 600×. Mitotic figures (arrow) were common, and nuclei had a moderate degree of nuclear atypia. **(G)** Congo red, 20×. Patchy areas of the extracellular material are congophilic with red staining, consistent with amyloid (arrows). **(H)** Congo red, 400×. Congophilic extracellular amyloid stains red. **(I)** Masson's trichrome, 20×. Much of the extracellular material is bright blue with trichrome, consistent with collagen matrix. **(J)** Masson's trichrome, 400×. The amyloid deposits (*) are pale blue while collagen fibers are bright blue (arrows).

Images of the thorax, however, revealed a soft tissue attenuating, mildly heterogeneously contrast-enhancing mass, surrounding the distal aspect of the lobar bronchus at the most cranioventral aspect of the right cranial lung lobe. The mass appeared rounded, measuring 25.6 mm (H) × 21.4 mm (W) × 19.1 mm (L), partially bordered by the lobar vessels and a rim of enhancement. A small mineral-attenuating focus was present at the dorsomedial aspect of the mass (Figure 3A). At the cranioventral aspect of the left cranial lung lobe, immediately ventral to the distal aspect of the lobar bronchus was an irregularly shaped nodule of mixed mineral and soft tissue attenuation. The soft tissue portion of the nodule was contrast-enhancing. The nodule measured approximately 10 mm (W) × 9.1 mm (H) × 11.5 mm (L) (Figure 3B). No other discrete pulmonary nodules were identified.

The right cranial pulmonary mass was concerning for a primary pulmonary neoplasm. The left cranial pulmonary nodule was more consistent with a granuloma given the degree of mineralization and was not considered consistent with a metastatic nodule. Aspirates of the right and left cranial lung lobe masses were non-diagnostic due to poor cellularity.

For definitive diagnosis and treatment, a thoracoscopic right cranial lung lobectomy was performed and the lobe was submitted for histopathology. Histologically, the lung mass was well demarcated and composed of polygonal epithelial cells arranged in sheets and cords with abundant eosinophilic extracellular material (Figures 3C,D). In a few areas, neoplastic epithelial cells had ghost cell morphology with keratin (not shown). The lung tumor had areas of necrosis (Figure 3E), and the epithelial cells demonstrated moderate nuclear atypia with a mitotic count of 40 figures per 10 high-power fields (2.37 mm^2^) (Figure 3F). A Congo red stain demonstrated patchy areas of congophilic material (amyloid) that were in small aggregates (Figures 3G,H). Larger deposits were not congophilic but stained bright blue with Masson's trichrome (Figures 3I,J). Areas of continuity from one type of material to another suggested that the amyloid (congophilic and pale blue with trichrome) was transformed to (or replaced by) a collagenous matrix material that resembled osteoid (not congophilic and bright blue with trichrome) and coalesced into larger trabeculae. Histopathology of the lung mass was interpreted as a metastatic amyloid-producing odontogenic tumor that closely resembled both oral tumors with the exception that the lung tumor had a greater amount of osteoid-like matrix.

Three weeks after treatment for the lung lesion, a bilateral rostral mandibulectomy was performed from the mesial aspect of the mandibular second premolars (306, 406) to treat the mandibular APOT with 1-cm gross surgical margins. Histopathology findings were identical to the aforementioned biopsy, and the rostral mandibulectomy margins were complete. The medial retropharyngeal lymph nodes and mandibular lymph nodes were also removed bilaterally, and histological evaluation found no evidence of metastasis.

All pathologic samples, including the initial specimen from 6 years prior, were sent to a single pathologist for review (CB). A pathologic review confirmed all specimens to be consistent with APOT with the primary difference being a higher mitotic rate in the initial oral tumor (25 mitotic figures per 10 high power fields, hpf) and metastatic lung tumor (40 per 10 hpf) vs. the second oral tumor (0 per 10 hpf). The dog is doing well with good oral function 9 months after mandibulectomy.

## Discussion

To the authors' knowledge, this is the first case report of a metastatic APOT in a canine. What is especially unusual in this case is that the dog had APOT in two separate locations in the oral cavity that presented 6 years apart. It is not possible to know which of these was the primary tumor that metastasized to the lungs. While the two oral tumors had similar histological features, the first had a higher mitotic rate and was larger. In retrospect, this tumor may have represented an APOT-like carcinoma as recently described in a dog (10). Although less likely, the first oral tumor potentially metastasized both to the lung and within the oral cavity.

Although rare, histologically benign epithelial odontogenic tumors are known to carry a metastatic potential in humans. Ameloblastoma has a biological subset referred to as the metastasizing ameloblastoma, which is new to the 2017 WHO classification of odontogenic tumors (12). This biologic subtype of ameloblastoma is diagnosed in retrospect, i.e., at the time that metastasis is confirmed, since histopathology of the primary tumor is generally benign without atypia that might suggest malignant behavior. Due to this biologic subtype, ameloblastoma in humans is classified as benign yet carries an ~2% metastatic potential (12).

New evidence on oncogenesis of canine ameloblastoma supports that ameloblastoma in dogs develops similarly to its human counterpart via mutations in the MAPK pathway (13). Thus, given the similar biologic oncogenetics between canine and human ameloblastomas, this tumor most likely also carries a small metastatic potential within the canine population. Metastasis most likely has never been documented due to the median age of survival after diagnosis in dogs, as well as the lack of follow-up staging post-treatment of these “benign” tumors. As APOT may actually represent a subset of ameloblastoma, it is feasible that these also have similar oncogenetics to human ameloblastomas and, as documented within this case report, carry a small metastatic potential.

Additional studies are necessary to better characterize the tumor (or group of tumors) that are currently known as APOT in animals. Since first described in animals, tumors of odontogenic epithelium that produce amyloid have fit poorly into the nomenclature for odontogenic tumors of both humans and animals. The term APOT was proposed to address this problem (3). For over 25 years, the diagnosis of APOT has allowed veterinary clinicians and pathologists to communicate effectively. However, it may be time to refine the diagnostic criteria and nomenclature. In humans, amyloid may be present in several types of odontogenic tumors and cysts; we expect that the same is true in animals. A recent report of APOT with histological characteristics of a carcinoma (10) and this case report of metastatic APOT suggest that there is biological variation within the group of tumors that fit the diagnostic criteria of APOT. Only after we have a better understanding of the clinical and histological variation of amyloid-producing odontogenic tumors can there be an updated classification that allows for a more precise diagnosis and more accurate prognostication.

Until this work can be performed, it is recommended that clinicians discuss the potential, albeit low, for distant metastasis in patients with APOT, especially if there are histological features of potential malignancy (i.e., frequent mitotic figures, nuclear atypia). There is also justification for long-term follow-up and monitoring since this patient was initially treated as a juvenile with metastasis becoming apparent 6 years later. A protracted time course of metastasis is similar to that reported in humans with metastatic ameloblastoma (14).

## Data Availability Statement

The original contributions presented in the study are included in the article/supplementary material, further inquiries can be directed to the corresponding author/s.

## Ethics Statement

Ethical review and approval was not required for the animal study because this case report was a clinical case and not a part of a research study. Written informed consent was obtained from the owners for the participation of their animals in this study.

## Author Contributions

CBW contributed to the clinical and surgical case management and completed the first manuscript draft. CMB provided the processing and interpretation for all histological samples. SG contributed to clinical and surgical case management and manuscript preparation. All authors assisted in writing and editing of the manuscript.

## Conflict of Interest

The authors declare that the research was conducted in the absence of any commercial or financial relationships that could be construed as a potential conflict of interest.
